# Research trends and hotspots of post-stroke dysphagia rehabilitation: a bibliometric study and visualization analysis

**DOI:** 10.3389/fneur.2023.1279452

**Published:** 2023-12-08

**Authors:** Yuanyuan He, Xuezeng Tan, Huiqi Kang, Huan Wang, Yuyao Xie, Dongxiang Zheng, Chen Li

**Affiliations:** ^1^College of Nursing, Jinan University, Guangzhou, China; ^2^Department of Critical Care Medicine, Hainan Hospital of Chinese PLA General Hospital, Sanya, China; ^3^Department of Neurology and Stroke Center, The First Affiliated Hospital, Jinan University, Guangzhou, China

**Keywords:** stroke, deglutition disorders, bibliometrics, rehabilitation, CiteSpace, VOSviewer

## Abstract

**Background:**

Post-stroke dysphagia (PSD) is one of the most prevalent stroke sequelae, affecting stroke patients’ prognosis, rehabilitation results, and quality of life while posing a significant cost burden. Although studies have been undertaken to characterize the pathophysiology, epidemiology, and risk factors of post-stroke dysphagia, there is still a paucity of research trends and hotspots on this subject. The purpose of this study was to create a visual knowledge map based on bibliometric analysis that identifies research hotspots and predicts future research trends.

**Methods:**

We searched the Web of Science Core Collection for material on PSD rehabilitation research from its inception until July 27, 2023. We used CiteSpace, VOSviewer, and Bibliometrix R software packages to evaluate the annual number of publications, nations, institutions, journals, authors, references, and keywords to describe present research hotspots and prospective research orientations.

**Results:**

This analysis comprised 1,097 articles from 3,706 institutions, 374 journals, and 239 countries or regions. The United States had the most publications (215 articles), and it is the most influential country on the subject. “Dysphagia” was the most published journal (100 articles) and the most referenced journal (4,606 citations). Highly cited references focused on the pathophysiology and neuroplasticity mechanisms of PSD, therapeutic modalities, rehabilitation tactics, and complications prevention. There was a strong correlation between the terms “validity” and “noninvasive,” which were the strongest terms in PSD rehabilitation research. The most significant words in PSD rehabilitation research were “validity” and “noninvasive brain stimulation,” which are considered two of the most relevant hotspots in the field.

**Conclusion:**

We reviewed the research in the field of PSD rehabilitation using bibliometrics to identify research hotspots and cutting-edge trends in the field, primarily including the pathogenesis and neurological plasticity mechanisms of PSD, complications, swallowing screening and assessment methods, and swallowing rehabilitation modalities, and this paper can provide in the follow-up research in the field of PSD rehabilitation. The results of this study can provide insightful data for subsequent studies in the field of PSD rehabilitation.

## 1 Introduction

According to a 2019 Global Burden of Disease Study research, stroke is still the second largest cause of mortality (11.6% of total deaths) and the third major cause of disability (1). Post-stroke dysphagia (PSD) is the most prevalent post-stroke complication, occurring between 37 and 78% of the time, and is difficult to recover from during a stroke (2). In accordance with studies, patients with dysphagia have a higher risk of aspiration and pneumonia than non-dysphagic patients (3, 4), resulting in lengthier hospitalization and an increased risk of death (5). Furthermore, qualitative research revealed that dysphagia has a negative impact on patients’ mental health, social communication, and family roles, in addition to modifying their physical functioning and eating habits (6). Early discovery, evaluation, and rehabilitation can lower the risk of hypoxia, lung infection, malnutrition, and other complications (7), Early screening is thus the first step in dysphagia rehabilitation; however, many clinical swallowing screens have high sensitivity but low specificity, and the accuracy of bedside swallowing screening tools to identify dysphagia in the acute phase of stroke remains unknown (8).

Neurostimulation of the pharyngeal motor cortex is the focus of neurological rehabilitation of swallowing function, with techniques separated into peripheral sensory and central stimulation (9). The use of drugs such as transient receptor potential (TRP) channel agonists and retropharyngeal or transcutaneous electrical stimulation (TES) methods to boost sensory input to improve oropharyngeal swallowing responses is referred to as peripheral stimulation. Transcranial direct current stimulation (tDCS) and repetitive transcranial magnetic stimulation (rTMS) are among the more frequently utilized modalities of central stimulation for the treatment of PSD (10, 11). Notably, speech and language pathology have significance in the rehabilitation of individuals with dysphagia (12).

Bibliometric analysis is useful in defining the current state of many research fields, as well as the scientific accomplishments of researchers, institutions, and countries, as well as potential research hotspots (13). Bibliometric analyses of global healthy eating, ophthalmology, and the application of artificial intelligence in diabetic retina by a number of researchers have offered strong insights into study topics of interest (14–16). The rehabilitation of PSD has garnered extensive attention in recent years, however, the research hotspots and upcoming research paths have yet to be clarified. As a result, we conducted a bibliometric analysis of publications on PSD rehabilitation from the Web of Science Core Collection (WoSCC) database during the last 25 years. Bibliometric analysis software is used to explore countries, institutions, journals, authors, references, and keywords, to learn about countries, journals, institutions, and authors with top influence in the research field; to acquire about burning references and hot keywords, to form a clustering theme of research in the field of PSD rehabilitation, to create understandable visual models and analyze current research trends and hotspots to reveal the evolution and development of the research area and anticipate future research directions. This study not only provides authors and researchers with an overall visual knowledge map and significant insights into the topic of PSD rehabilitation, but it additionally provides meaningful references for future research.

## 2 Materials and methods

### 2.1 Search strategy and data collection

**Figure 1:**
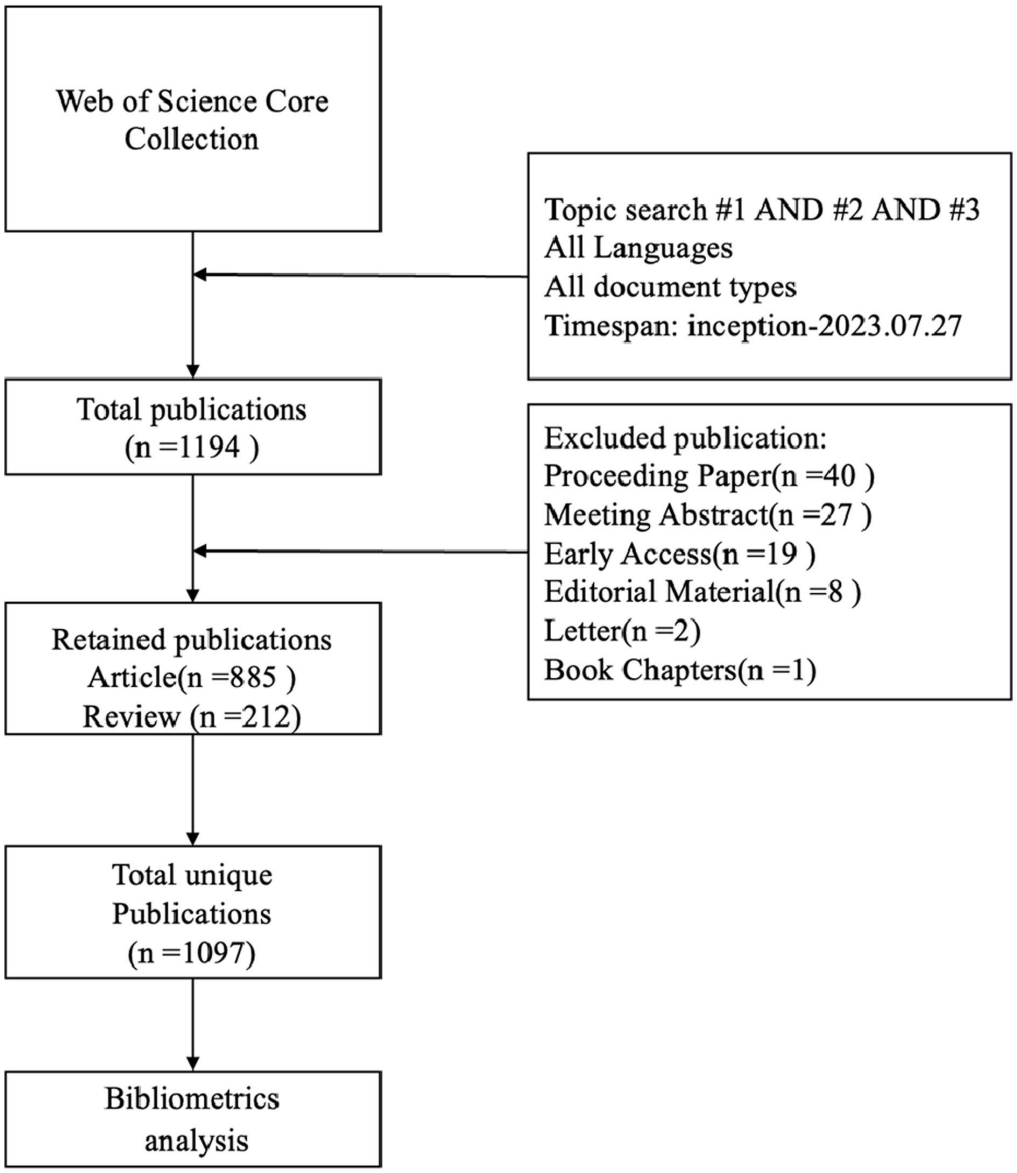
Flow chart of literature selection.

Web of Science Core Collection (WoSCC) is a globally influential, authoritative, and comprehensive database that provides comprehensive citation data covering multiple disciplines and has a unique advantage in conducting multidisciplinary and international bibliometric analyses (17); thus, we chose WoSCC as the source database for data retrieval in this research. The data retrieval strategy is as follows: TS = (stroke OR apoplexy OR “cerebrovascular accident” OR “brain vascular accident” OR “cerebral hemorrhage” OR encephalorrhagia OR “cerebral ischemia”) AND TS = (“Deglutition Disorder” OR “Swallowing Disorder*” OR Dysphagia OR “Oropharyngeal Dysphagia” OR “Esophageal Dysphagia”) AND TS = (rehabilitation OR recovery), The search period was set to July 27, 2023, and the genre of material was limited to articles and reviews. Irrelevant material was eliminated by skimming the titles and abstracts, which included Proceeding Papers, Meeting Abstracts, Letters, and so on. Finally, 1,097 articles were found to satisfy the inclusion criteria. The flow chart of the literature screening is shown in Figure 1.

### 2.2 Data analysis

We selected articles published within the last 25 years so that we could not only analyze the literature’s development cycle and gain insight into how the impact of the literature has changed over time, which would be useful for researchers interested in the field of PSD rehabilitation to understand the history of the field and its evolutionary trends but also identify the literature that has had a key impact on the field of study at each stage of its development and authors. We also uncover research hotspots and trends in the field of PSD rehabilitation by using bibliometric analysis algorithms to find references with the highest citation bursts, keywords, and keyword clustering.

The steps for bibliometric analysis are as follows: We used VOSviewer 1.6.19 (Leiden University, Netherlands), CiteSpace 6.1.R6 (Drexel University, PA, United States), and the Bibliometrix R package (4.1.3) to analyze the included literature. The main contents of the analysis include countries, institutions, journals, authors, references, and keywords. Data was processed using Excel.

## 3 Results

### 3.1 Analysis of publication outputs

**Figure 2:**
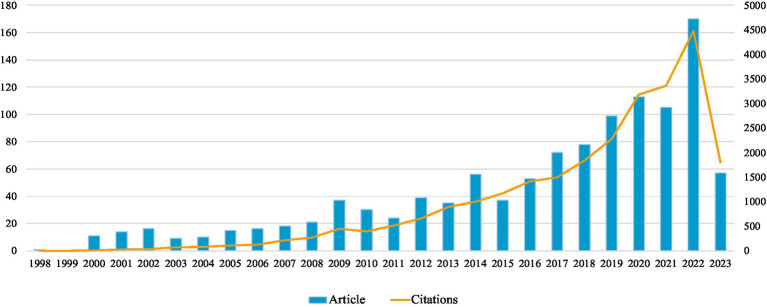
Annual publication trend of PSD rehabilitation.

The analysis contains 1,097 papers published between 1998 and 2023, containing 885 articles and 212 reviews. The total h-index is 69, the total number of citations is 24,141, and the total number of frequently referenced articles is 21.99. Figure 2 depicts the annual publication and citation counts for PSD rehabilitation, which shows a generally consistent but unstable growth trend, with a slow increase in the number of publications between 1998 and 2013, ranging from about 10 to 40 articles per year, and then an accelerated growth beginning after 2014, except a slight decline in the number of publications in 2015 and 2021. The overall trend is upward and will peak in 2022, indicating that researchers are focusing more on PSD rehabilitation.

### 3.2 Country/region and institution contributions

**Figure 3:**
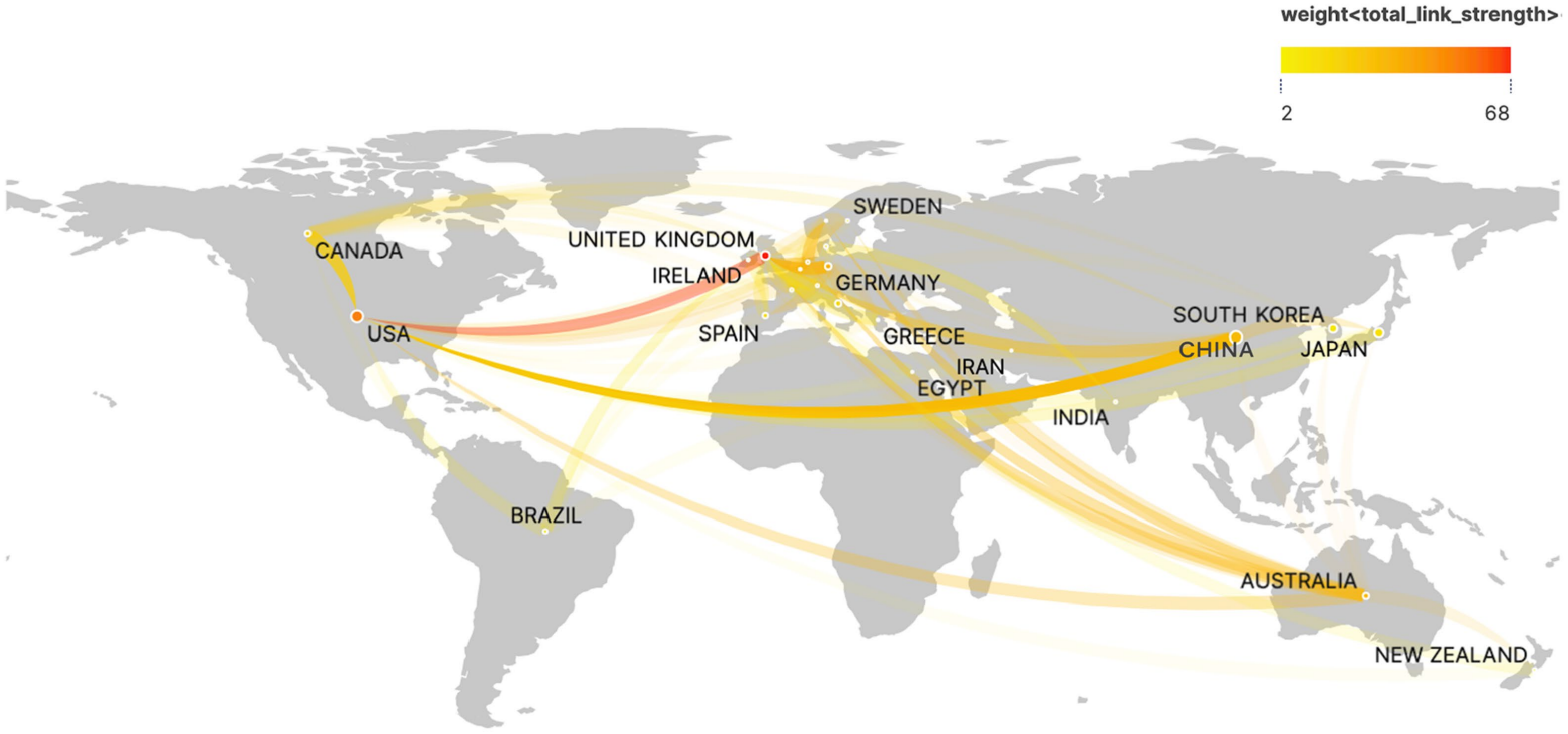
Network of international cooperation of PSD rehabilitation in geographic visualization.

PSD rehabilitation research is being conducted in 239 countries/regions by 3,706 institutions. The top 10 countries and institutions are given in Table 1 determined by the number of publications. The United States has the most publications and citations (193 articles, 7,994 citations), followed by China (186 articles, 1,555 citations), and Japan (131 articles, 2,034 citations), which account for nearly one-half the total number of publications in the field. Figure 3 depicts how a country cooperation network is used with VOSviewer and Scimago Graphica. The thickness and hue of the collaboration network connecting lines can show the degree of cooperation between countries or areas, with the closer the node color to red suggesting the intensity of the research collaboration. The analysis results indicate that the UK has the strongest international collaboration strength (with 68 total connection strengths), forming an extensive research collaboration network between the UK, the US, China, and Canada. The h-index is an estimate of a scholar’s or country’s scientific impact (18), and it balances the relationship between the number of published papers and their quality (the impact of the papers or the number of times they are cited) to provide a more complete picture of the impact of a scholar’s research results. An examination of the PSD science field’s h-index (Table 1) reveals that the United States has the greatest h-index (40) and total scientific research impact. The analysis of the PSD science field’s h-index (Table 1) reveals that the United States has the greatest h-index (40) and total scientific research impact. However, it is worth mentioning that, despite accounting for a small number of publications, the United Kingdom and Canada rank second only to the United States in terms of article citations and h-index, and their significance should not be overlooked. Additionally, while China ranked second in terms of publications, it ranked in the middle of the pack in terms of h-index (23) and article citations (1555), indicating that the academic impact of the field is limited and that high-quality research needs to be conducted to increase the academic impact of the field.

**Table 1:** Ranking of top 10 countries and institutions involved in the PSD rehabilitation field.

Rank	Country/region	Count (%)	Citations	H-index	Institution	Count (%)	Citations
1	USA	193 (17.78%)	7,994	40	N8 Research Partnership	46 (4.014%)	2,322
2	CHINA	186 (16.94%)	1,555	23	University of Manchester	36 (3.141%)	1913
3	JAPAN	131 (11.93%)	2034	24	The State University System of Florida	32 (2.79%)	2,160
4	SOUTH KOREA	97 (8.83%)	1,378	19	University of Florida	26 (2.27%)	2098
5	UNITED KINGDOM	92 (8.38%)	4,336	32	University of London	26 (2.27%)	1926
6	GERMANY	58 (5.28%)	1,444	21	Kumamoto Rehabillitation Hospital	23 (2.01%)	528
7	ITALY	56 (5.10%)	1,039	18	Seoul National University	21 (1.83%)	522
8	AUSTRALIA	49 (4.46%)	826	17	University College London	20 (1.75%)	1,276
9	CANADA	43 (3.92%)	3,545	25	University of Toronto	20 (1.75%)	2,727
10	SPAIN	39 (3.55%)	1,008	16	Autonomouw University of Barcelona	19 (1.66%)	1,276

### 3.3 Institutional analysis

**Figure 4:**
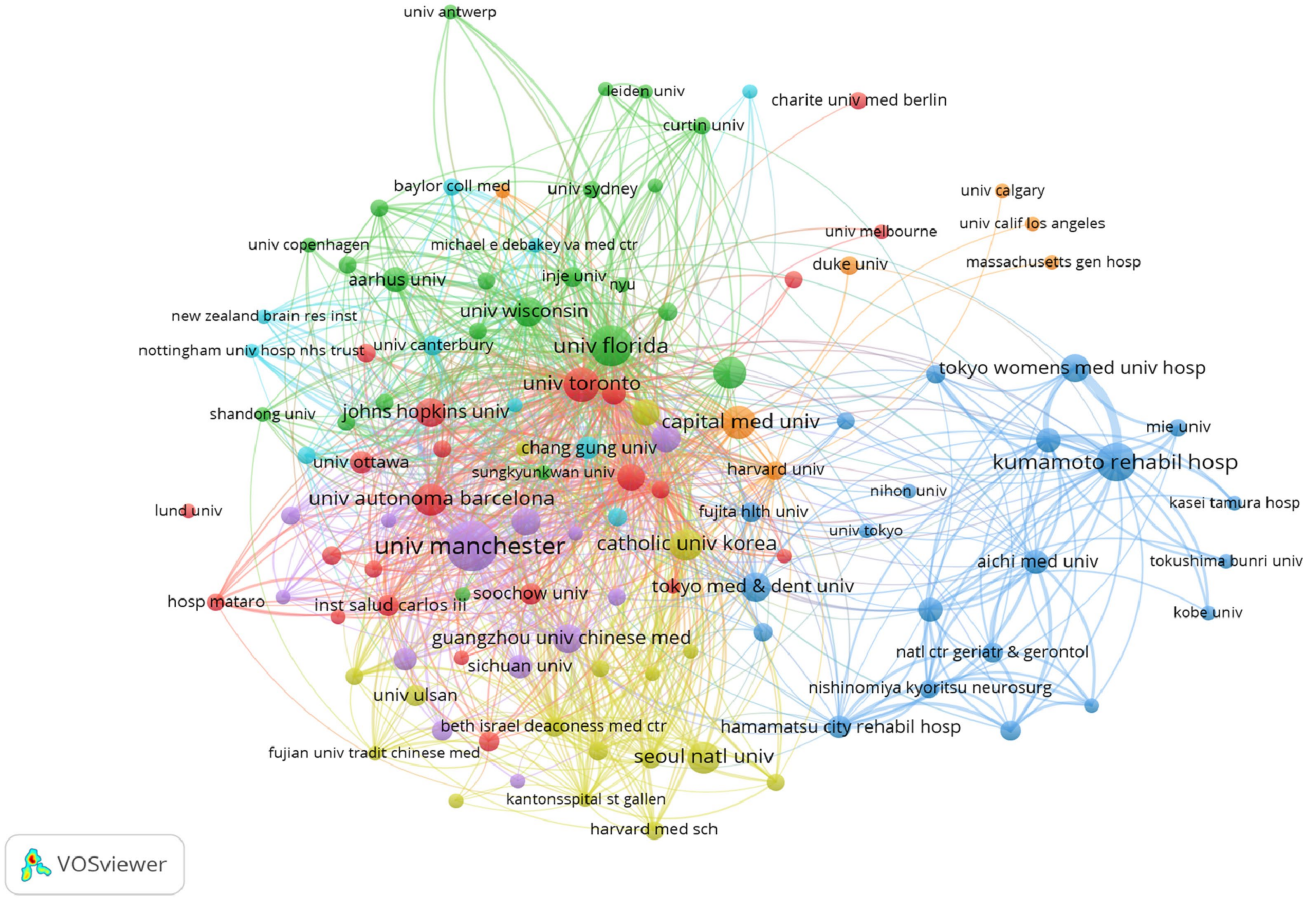
Institutional cooperation network diagram of PSD rehabilitation.

The analyzed search results in WoSCC show that N8 Research Partnership is the institution with the highest number of publications and citations (46 articles, 2,322 citations), followed by the University of Manchester (36 articles, 1,913 citations), The State University System of Florida (32 articles, 2,160 citations), and the University of Florida (26 articles, 2098 citations), most of these institutions originated from Europe and the United States, showing a wide range of research interests and a strong influence in the field. VOSviewer has been utilized to create a network of institutional collaborations (Figure 4), with 114 institutions identified as having published at least five articles, and seven color clusters formed based on the intensity of collaboration between institutions, indicating a broader research collaboration between the clusters, with the purple and green sections each containing 23 institutions.

### 3.4 Journal and co-cited journal distribution

**Figure 5:**
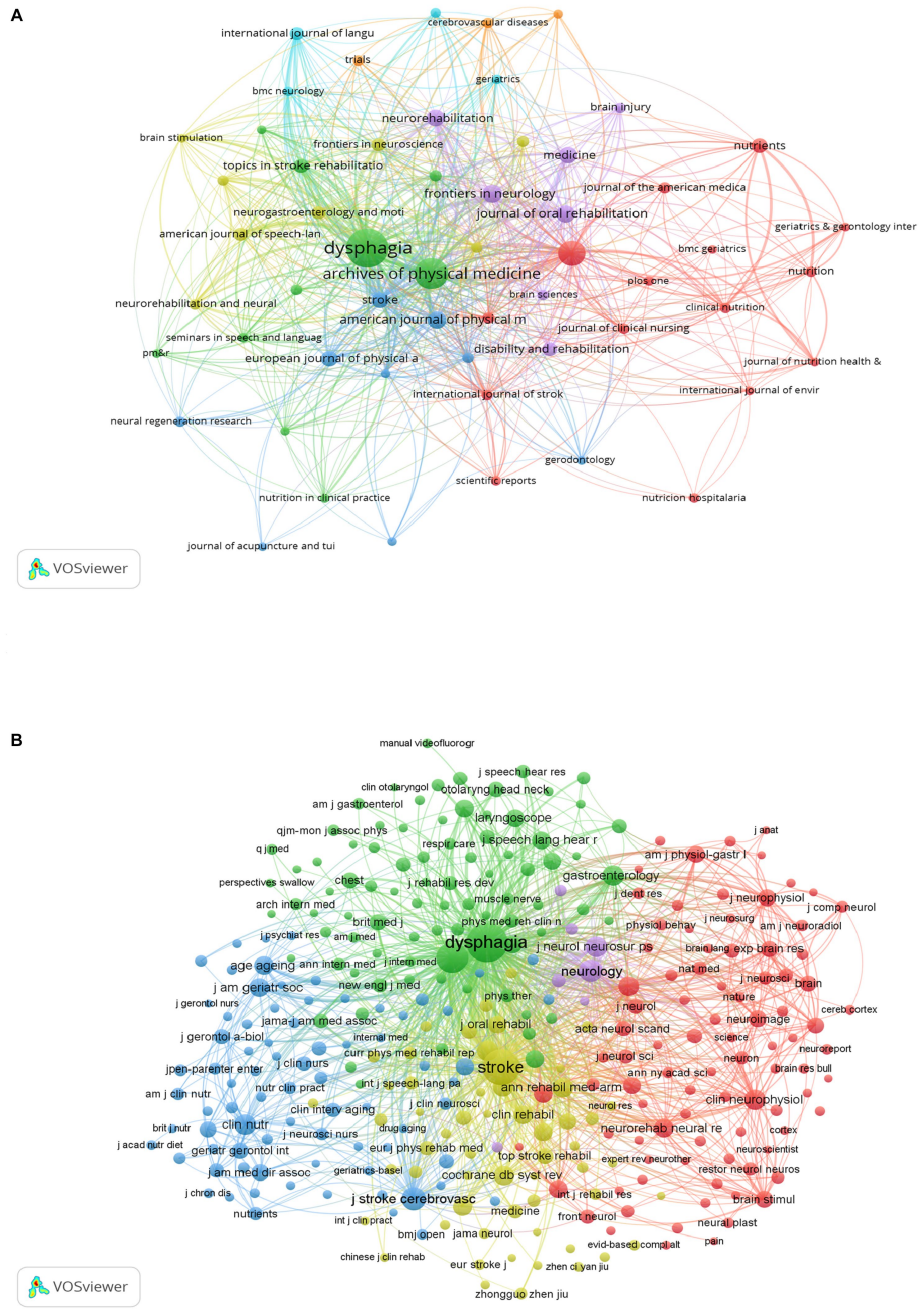
Analysis journals of PSD rehabilitation. **(A)** Journals co-occurrence analysis. **(B)** Co cited journals analysis.

Papers on PSD rehabilitation have been published in 374 scholarly publications since 1998. We used VOSviewer to perform a visual analysis of co-occurrence relationships between journals (Figure 5A) and co-citation relationships between co-cited journals (Figure 5B), and based on the results of the analysis, we plotted the top 10 journals and co-cited journals related to PSD rehabilitation (Table 2). Dysphagia took the top spot with 100 publications (8.73%), followed by the Archives of Physical Medicine and Rehabilitation (62 publications, 5.41%, 1,790 citations). Stroke (17 publications, 1.48%, 3,179 citations) was one of the top 10 journals with the highest impact. Stroke (17 articles, 1.1%, 3,179 citations) has the highest impact factor among the top 10 journals, as well as the most citations, demonstrating its strong academic importance in the subject of PSD rehabilitation. After analyzing the top 10 journals and co-cited journals, we discovered that six journals originated in the United States and four in the United Kingdom, which corresponds with the country/region and institutional contributions examined earlier and is sufficient to demonstrate the influence of these two countries in this research field.

**Table 2:** Top 10 journals and co-cited journals of PSD rehabilitation.

Rank	Journal	Count(%)	IF (2022)	JCR	Co-cited journal	Citations	IF (2022)	JCR
1	Dysphagia	100 (8.73%)	2.6	Q2	Dysphagia	4,606	2.6	Q2
2	Archives of Physical Medicine and Rehabilitation	62 (5.41%)	4.3	Q1	Stroke	3,179	8.3	Q1
3	Journal of Stroke and Cerebrovascular Diseases	42 (3.67%)	2.5	Q3	Archives of Physical Medicine and Rehabilitation	1790	4.3	Q1
4	Frontiers in Neurology	22 (1.92%)	3.4	Q3	Journal of Stroke and Cerebrovascular Diseases	721	2.5	Q3
5	American Journal of Physical Medicine and Rehabilitation	20 (1.75%)	3.0	Q1	Neuron	660	16.2	Q1
6	Journal of Oral Rehabilitation	20 (1.75%)	2.9	Q2	Gastroenterology	492	29.4	Q1
7	Neurorehabilitation	19 (1.66%)	2.0	Q3	Journal of Neurologic Physical Therapy	479	3.8	Q2
8	Medicine	18 (1.57%)	1.6	Q4	Lancet	474	168.1	Q1
9	European Journal of Physical and Rehabilitation Medicine	17 (1.48%)	4.5	Q2	Clinical Neurophysiology	441	4.7	Q2
10	Stroke	17 (1.48%)	8.3	Q1	Cochrane Database of Systematic Reviews	985	8.4	Q1

**Figure 6:**
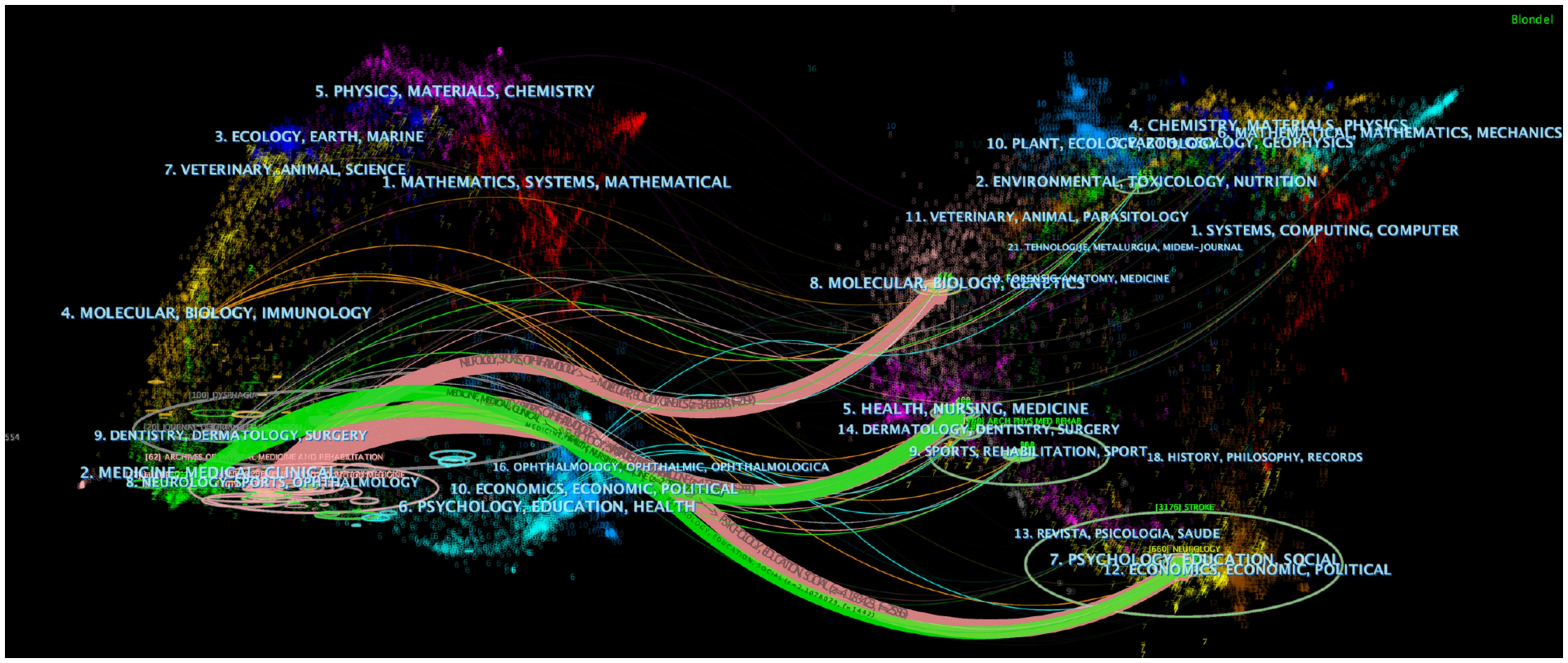
The dual map overlay of journals in PSD rehabilitation.

Following this, we used CiteSpace to analyze the included literature with a dual map overlay (Figure 6), which links the cited journals on the left to the cited journals on the right to provide a clear, visual interpretation of citations for various combinations of publications, allowing for the identification of citation relationships, subject distributions, and patterns of movement across multiple disciplines (19). The ellipses on the map depict the number of publications relating to journals and the ratio of authors to the number of publications. The length of the ellipse represents the number of authors, the width of the ellipse represents the number of publications, and the curves between the left and right sides of the map are citation links, the trajectories of which provide an interpretation of the field’s interdisciplinary relationships. The Z score thicker the links, the greater the score of the score function. Finally, we identified 5 major citation trajectories (pink and green), with publications in the fields of Neurology, Sports, and Ophthalmology (pink trajectory) influenced by publications in the domains of Molecular, Biology, and Genetics (Z = 3.46, *f* = 2,192), Health, Nursing, and Medicine (Z = 4.13, *f* = 2,560), and Psychology, Education, and Social (Z = 4.18, *f* = 2,586). Furthermore, past publications in the Health, Nursing, Medicine (Z = 2.79, *f* = 2,822) and Psychology, Education, and Social (Z = 2.11, *f* = 1,442) domains influenced publications in the Medicine, Medical, and Clinical (green track). Overall, this implies that the topic of PSD rehabilitation is strongly tied to basic scientific, clinical, nursing, and social subjects and that there is scope for multidisciplinary, collaborative study.

### 3.5 Authors and co-cited authors

**Figure 7:**
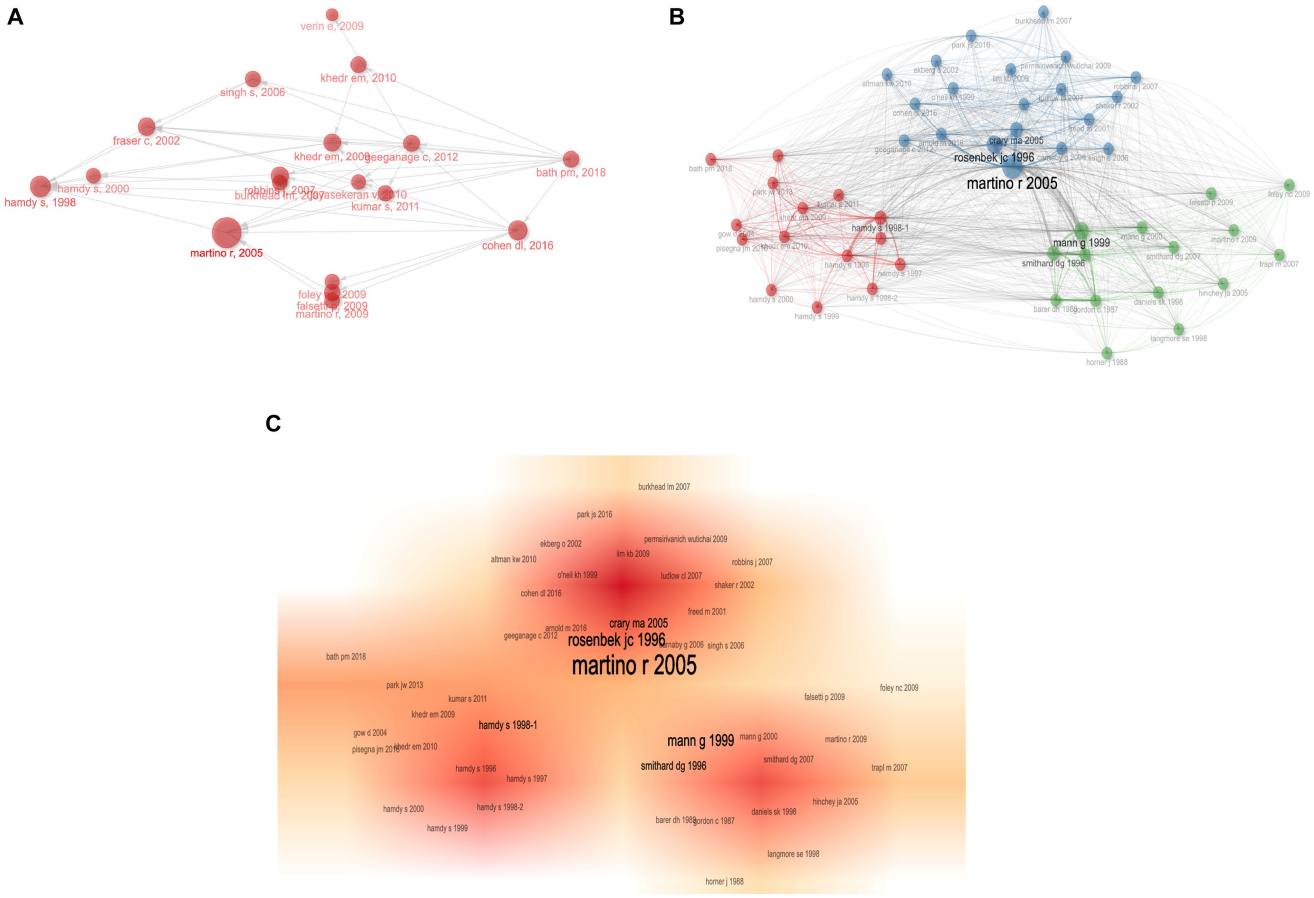
Co-authorship analysis of authors in PSD rehabilitation. **(A)** Historiography of influential author. **(B)** Network diagram of influential co-cited authors. **(C)** Influential co-cited author density visualization.

To ensure the precision of data analysis, we initially leveraged the ResearcherID and ORCID functionalities of Web of Science to discern each author. Both systems bestow a distinct identifier upon every author, effectively addressing issues stemming from name ambiguities. Subsequently, before inputting data into the bibliometric analysis software, we inspected the raw data using Excel. Variations of names that unmistakably belonged to the same author were manually consolidated. Through our analysis, it was determined that the PSD rehabilitation domain encompassed contributions from a total of 4,991 authors. For our analysis, we used the Bibliometrix R package (4.1.3) in conjunction with R version 4.3.1, which allowed us to access representative scholars in the field to understand core research trends (20). The top 10 authors and co-citing authors of PSD rehabilitation research are shown in Table 3. The author with the most publications is Hamdy S from the University of Manchester (35 papers, 3.00%, h-index = 20), followed by Wakabayashi H (26 articles, 2.37%, h-index = 12) and Yoshimura Y (23 articles, 2.09%, h-index = 11). Hamdy S is also the most cited author (545 citations), with Martino R (461 citations) and Smithard DG (374 citations) coming in second and third, respectively. Figure 7A depicts a timeline of prominent authors in the field of PSD rehabilitation research, with the larger red circle representing the scholar’s contribution to the discipline’s research area. Hamdy S is a key pioneer in the subject, and his study “Recovery of swallowing after dysphagia stroke relates to functional reorganization in the intact motor cortex” is an example of his work (21). This paper investigates the mechanisms of swallowing recovery in PSD, implying a role for intact hemisphere reconfiguration in recovery and laying the groundwork for future research areas in PSD rehabilitation. The author cooperation network diagram (Figure 7B) depicts collaboration and relationships between authors and co-citing authors in the field. The author collaboration network formed by combining Figures 7A,B demonstrates that Martino R from the University of Toronto plays an important role in bridging the gap between research in the field, as demonstrated by his work “Dysphagia after stroke: incidence, diagnosis, and pulmonary complications, “which determined the prevalence of dysphagia and associated lung function impairment in stroke patients (2). The study has raised concerns among PTSD researchers about the link between dysphagia and pneumonia. Bath PM is a co-author of a new study in this field, “Swallowing therapy for dysphagia in acute and subacute stroke,” which suggests that swallowing therapy may minimize hospitalization, dysphagia, and chest infections while also improving swallowing capacity. Chest infections, and may improve swallowing ability and provide new ideas for future swallowing therapy (22), providing new ideas for future swallowing therapy. Figure 7C shows that the thicker and larger the author’s name near the red colored block, the more influential that author is in the discipline, showing that Martino R is the most influential academic in this field of study.

**Table 3:** Top 10 authors and co-cited authors of PSD rehabilitation.

Rank	Author	Count (%)	H-index	Co-cited author	Citations
1	Hamdy S	35 (3.00%)	20	Hamdy S	545
2	Wakabayashi H	26 (2.37%)	12	Martino R	461
3	Yoshimura Y	23 (2.09%)	11	Smithard DG	374
4	Nagano F	17 (1.55%)	8	Crary MA	348
5	Shiraishi A	16 (1.46%)	10	Logemann JA	342
6	Bise T	15 (1.37%)	8	Daniels SK	332
7	Shimazu S	15 (1.37%)	8	Mann G	285
8	Michou E	14 (1.27%)	11	Rosenbek JC	260
9	Momosaki R	13 (1.18%)	8	Robbins J	255
10	Park JS	13 (1.18%)	9	Langmore SE	199

### 3.6 Analysis of research hotspots

#### 3.6.1 Publications with the highest number of citations

**Figure 8:**
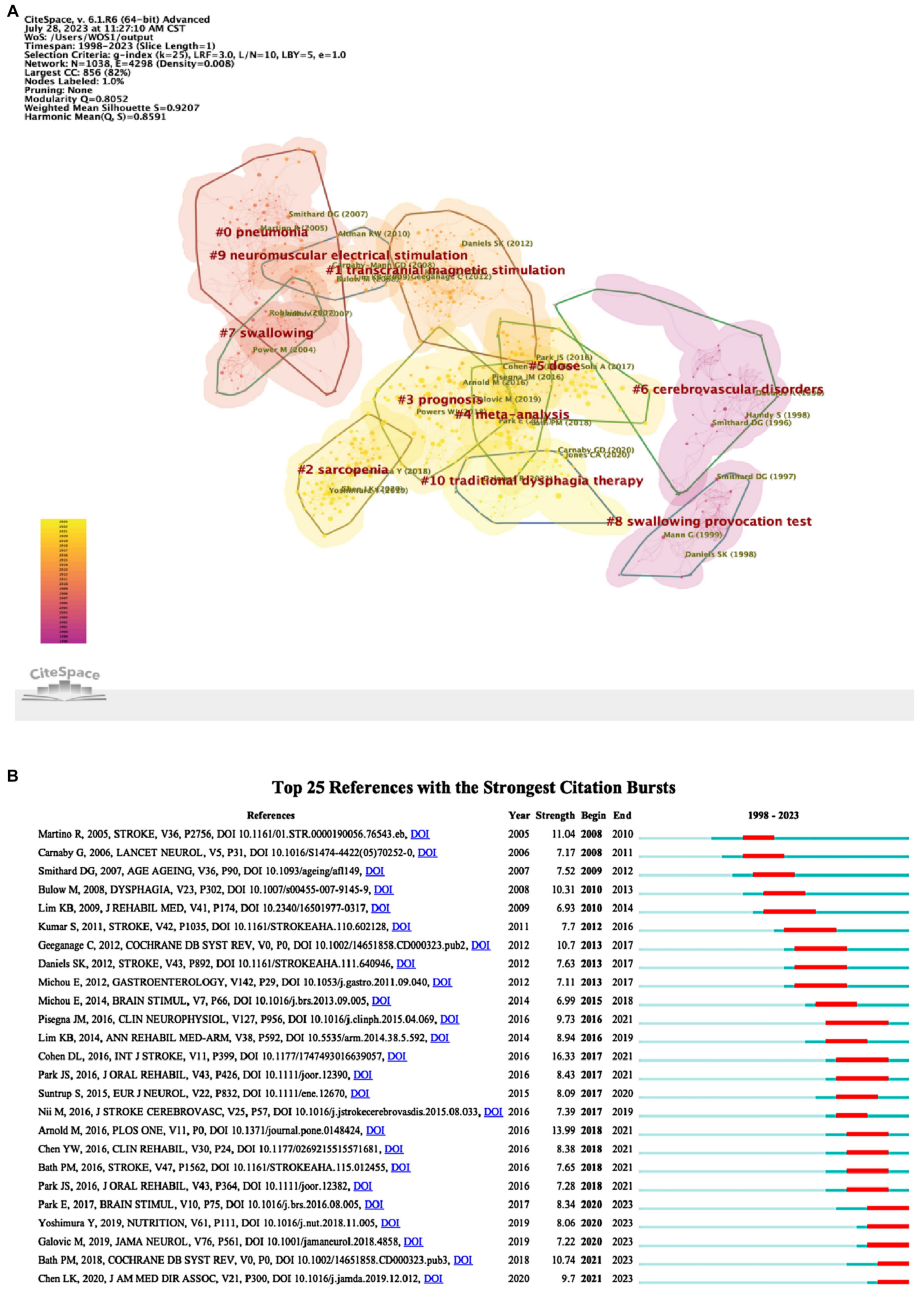
References analysis of PSD rehabilitation. **(A)** Co-citation references clustering. **(B)** Top 25 references with the strongest citation bursts.

Table 4 shows the top 10 most referenced references in the PSD rehabilitation study domain. Among these, “Dysphagia after stroke: incidence, diagnosis, and pulmonary complications” had the most citations (326), and it was written by Martino R, which correlates to the previous study’s findings. This matches the results of the preceding author’s contribution analysis. In addition to being the most cited document, “Interventions for dysphagia and nutritional support in acute and subacute stroke” had the highest centrality (Centrality = 0.08). This article focuses on analyzing the impact of swallowing therapy, feeding, nutrition, and fluid supplementation on functional outcomes and death in patients with acute or subacute stroke with dysphagia (23), and it informs future research on nutritional support for stroke patients. After reviewing the 10 cited references, we discovered that this research focused on PSD etiology and neuroplasticity processes, intervention approaches, rehabilitation tactics, and complication prevention. CiteSpace was used to cluster the co-cited references (Figure 8A), CiteSpace provides two indicators, the module value (Q value) and the average profile value (S value), which can be used to judge the effectiveness of the mapping, the Q value is generally in the interval of [0, 1], the Q > 0.3 represents the division of the structure of the significant, the S > 0.7, that is, the clustering reaches a high efficiency, that is, the results are credible. The grouping coefficients are Q = 0.8052, and S = 0.9207. The color change from purple to yellow represents the time dimension, signifying a shift in study focus and trend. As seen in the picture, the field has progressed from a focus on pneumonia to investigating transcranial magnetic stimulation for the rehabilitation of PSD and is now concentrating on the prognosis of PSD, intervention dose, and dysphagia treatment.

**Table 4:** Top 10 co-cited references of PSD rehabilitation.

Rank	References	Cited frequency	Centrality	Document title	Source	IF (2022)	JCR
1	Martino et al. (2)	326	0.02	Dysphagia after stroke: incidence, diagnosis, and pulmonary complications.	Stroke	8.3	Q1
2	Crary et al. (24)	189	0.03	Initial psychometric assessment of a functional oral intake scale for dysphagia in stroke patients.	Archives of Physical Medicine and Rehabilitation	4.3	Q1
3	Hamdy et al. (21)	107	0.01	Recovery of swallowing after dysphagic stroke relates to functional reorganization in the intact motor cortex.	Gastroenterology	29.4	Q1
4	Cohen et al. (25)	84	0.06	Post-stroke dysphagia: A review and design considerations for future trials.	International Journal of Stroke	6.7	Q1
5	Robbins et al. (26)	70	0.06	The effects of lingual exercise in stroke patients with dysphagia.	Archives of Physical Medicine and Rehabilitation	4.3	Q1
6	Fraser et al. (27)	66	0.02	Driving plasticity in human adult motor cortex is associated with improved motor function after brain injury.	Neuron	16.2	Q1
7	Khedr et al. (28)	65	0.04	Treatment of post-stroke dysphagia with repetitive transcranial magnetic stimulation.	Acta Neurologica Scandinavica	3.5	Q2
8	Geeganage et al. (23)	57	0.08	Interventions for dysphagia and nutritional support in acute and subacute stroke.	Cochrane Database of Systematic Reviews	8.4	Q1
9	Falsetti et al. (29)	55	0	Oropharyngeal dysphagia after stroke: incidence, diagnosis, and clinical predictors in patients admitted to a neurorehabilitation unit.	Journal of Stroke and Cerebrovascular Diseases	2.5	Q3
10	Bath et al. (22)	54	0.01	Swallowing therapy for dysphagia in acute and subacute stroke.	Cochrane Database of Systematic Reviews	8.4	Q1

#### 3.6.2 Analysis of reference citation bursts

Using CiteSpace to identify the references with the strongest citation bursts can be used to anticipate future research frontiers. Figure 8B depicts the top 25 references that elicited the most powerful bursts, with the duration of the burst shown in red. “Post-stroke dysphagia: A review and design considerations for future trials” was the literature with the highest burst citation strength (strength = 16.33), and it focused on PSD pathogenesis, diagnosis, dysphagia management, pharmacologic treatment, and post-stroke pneumonia prevention from 2017 to 2021. Moreover, we discovered 5 co-cited references in the recent outbreak phase (22, 30–33). These 5 co-cited references in the recent outbreak are primarily concerned with the topics of pathogenesis and neuroplasticity mechanisms of PSD, swallowing treatment, and rehabilitation effects.

#### 3.6.3 Clustering analysis and keyword occurrence frequency

**Figure 9:**
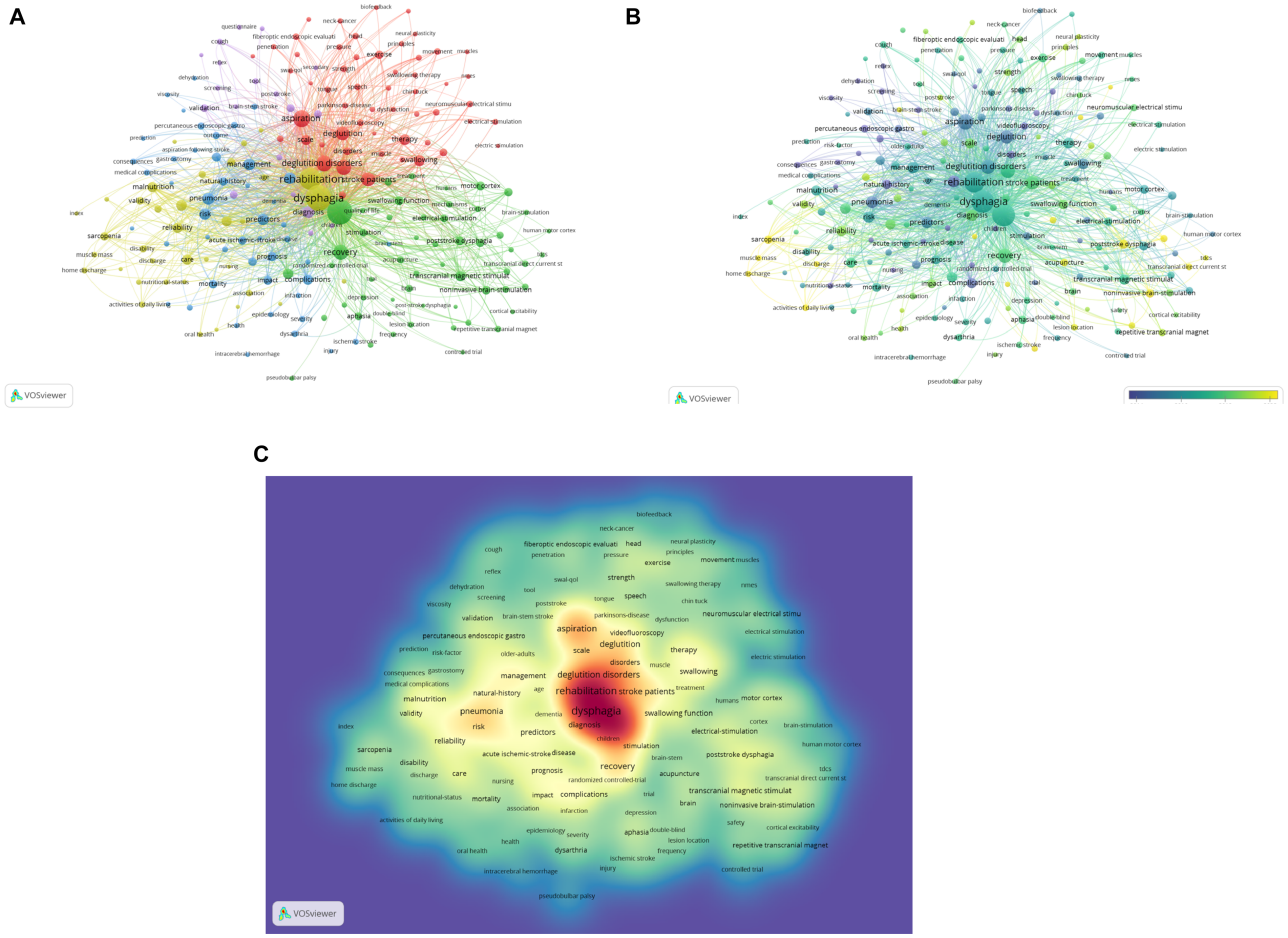
Keywords analysis of PSD rehabilitation. **(A)** Cluster view of keywords. **(B)** Time-overlapping visualization of keywords. **(C)** The density visualization map of keywords.

The term cluster analysis can summarize research topics and aid in understanding research hotspots in the subject. VOSviewer estimated 3,250 keywords, and the terms with a frequency of occurrence higher than 5 times or more were removed and incorporated in the co-occurrence analysis, resulting in 5 color clusters (Figure 9A), which indicate 5 study directions. In addition to “dysphagia” and “rehabilitation,” the main keywords in the yellow clusters also included “malnutrition “, “reliability,” “validity,” and “care”; the main keywords in the green cluster are “quality of life,” “recovery,” “stimulation,” “transcranial magnetic stimulation” and “noninvasive brain-stimulation”; in the red cluster, the main keywords were “deglutition disorders,” “aspiration,” “deglutition,” “scale,” “therapy”; the main keywords in the blue cluster are “management,” “pneumonia,” “predictors,” “risk”; the main keywords in the purple cluster are “diagnosis “, “validation,” “tool,” “brain-stem stroke.” Except for “dysphagia,” “rehabilitation,” and “deglutition disorders,” the terms “diagnosis,” “pneumonia,” “risk,” and “aspiration” had the most weight, according to the keyword density map (Figure 9B). We also created a temporal overlap representation of the keywords (Figure 9C), in which the gradient from purple to yellow represents the period of the keywords, with yellow signifying current occurrences of the keywords, which represent recent periods of study in the subject.

#### 3.6.4 Keyword citation burst analysis and trend topics

**Figure 10:**
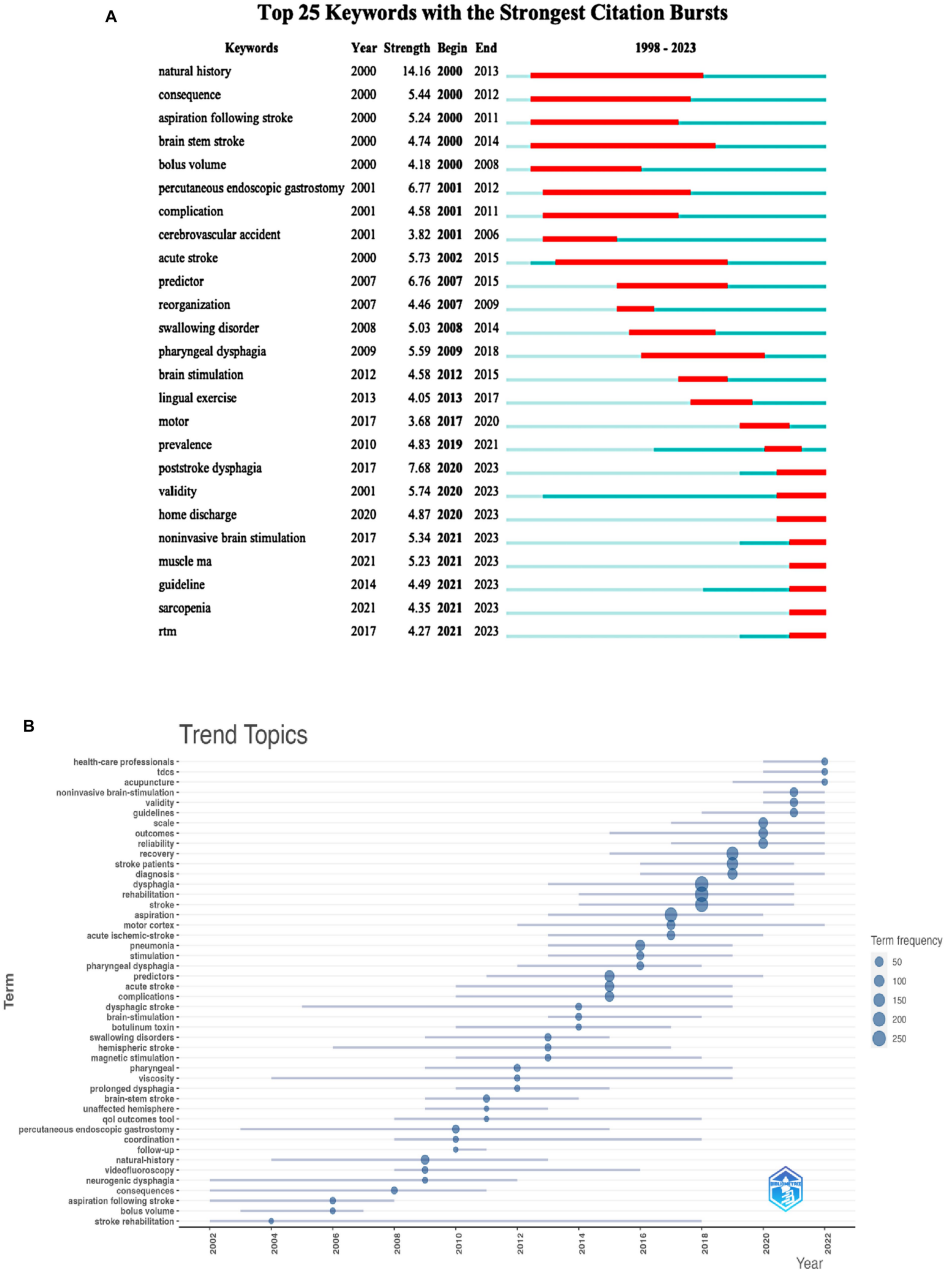
Keywords analysis of PSD rehabilitation. **(A)** Top 25 keywords with the strongest citation bursts. **(B)** Trend topics of keywords.

Burst keywords are high-frequency keywords that erupt at a specific moment, showing the emergence of hotspots in the study area and anticipating research trends. Figure 10A depicts the top 25 terms with the highest number of citations, with “natural history,” “percutaneous endoscopic gastrostomy,” and “predictor” being the top three keywords with the most citation numbers. Furthermore, the terms that received the most attention were “consequence” (2000–2012), “brain stem stroke” (2000–2014), “acute stroke” (2002–2015), and “complication” (2001–2011). Excluding words related to the search term, “validity,” “noninvasive brain-stimulation,” “home discharge,” “muscle mass,” “guideline,” and “sarcopenia,” “rTMS” are the keywords of citation explosion during 2020–2023, which are regarded as the hotspots that researchers pay high attention to shortly, among which “validity” and “noninvasive brain-stimulation” are the keywords. Among these, “validity” and “non-invasive brain stimulation” are the most powerful. We also used Bibliometrix R to look for trending themes in the field of PSD rehabilitation (Figure 10B). Keywords like “health care professionals,” “transcranial direct current stimulation (tDCS),” and “acupuncture” are suggestive of future research trends. Overall, research hotspots in the field of PSD rehabilitation focus on PSD-related guidelines, the accuracy, and validity of screening tools, noninvasive brain stimulation, transcranial magnetic stimulation (rTMS), tDCS, and acupuncture for PSD rehabilitation.

## 4 Discussion

### 4.1 General information

In this study, we used CreateSpace, VOSviewer, and Bibliometrix R to assess the literature on PSD rehabilitation. The results of the analysis show that the trend of growth in the number of annual publications over the last 25 years has been divided into two phases, with slow growth in the number of publications between 1998 and 2013, a period that may be related to the increased focus of research in the field on the incidence of stroke and the treatment of the disease. The number of publications begins to increase between 2014 and 2023, with the most pronounced trend of growth in 2022, which is likely to be explained by Guidelines issued by the European Stroke Organization and the European Swallowing Society on the diagnosis and treatment of post-stroke dysphagia (34). The guideline proposes the impact of PSD on stroke outcomes, nutritional screening for dysphagia, dysphagia assessment, and PSD treatment, and it provides evidence-based recommendations for the management of PSD by a multidisciplinary team aimed at preventing, diagnosing, and treating PSD, as well as bringing PSD rehabilitation to the attention of a broader range of researchers and providing ideas for future studies.

The examination of national and institutional collaboration networks reveals that research in the field of PSD rehabilitation dominates in industrialized nations. On the one hand, this may be attributable to the significance that industrialized nations have on PSD rehabilitation, and on the other, it may be connected to the level of the national economy, policy, and financial assistance. The United States has not just the most publications, but also the most citations. The United Kingdom does not have the most publications, but it is second only to the United States in terms of citations and the h-index, and it is one of the top countries in terms of total institutional effect and worldwide influence. China has the most common stroke cases in the world (35), and as a result, it places a high priority on stroke-related research, which explains why China comes second in terms of the number of publications. However, while China has a high number of publications, its citation, and h-index rankings are moderate, indicating that the academic impact has not been matched and that, in the future, it should focus on innovation and high-quality research in the subject area to improve the quality of articles.

According to the findings of this investigation, Dysphagia is the journal with the most articles and citations. Except for three journals that are Q1, the majority of the top 10 are Q2 or Q3, with an average IF of 3.51, showing that the field’s academic influence still needs to be enhanced. According to the findings of the study, Hamdy S was not only the author with the most publications, but also the author with the highest co-citation rating. The primary emphasis of this author’s study was on the mechanics of swallowing recovery in PSD (21), which provided a solid foundation for further research on PSD rehabilitation. The second most prominent author in the area is Martino R., whose work first revealed the association between PSD and the prevalence of pneumonia (2), promoting researchers in the field to re-examine PSD and perform in-depth research on PSD and pneumonia.

The studied search results in WoSCC revealed that papers on PSD rehabilitation include a wide range of topic areas, with the top three being Rehabilitation (259 articles), Clinical Neurology (225 articles), and Neurosciences (206 articles). Despite the limited number of papers (78), Peripheral Vascular Disease has the largest amount of single citations (58.95). In addition, the papers include Otorhinolaryngology, General Internal Medicine, Peripheral Vascular Disease, Sports Sciences, Nutrition Dietetics, and Audiology Speech Language Pathology. Furthermore, a biplot overlay analysis of published journals reveals that PSD rehabilitation is influenced by basic scientific, clinical, nursing, and social subjects, implying that PSD rehabilitation is a complex area of research requiring multidisciplinary collaboration and intervention implementation.

### 4.2 Research hotspots and trends

First, reference co-citations and keyword co-occurrences are investigated in this study to discover research topics and trends in the field of PSD rehabilitation. Second, the findings of temporal overlap visualization and keyword burst detection were utilized to highlight research hotspots and frontiers in the field. Finally, the field’s hotspots and cutting-edge developments were highlighted, covering the pathophysiology and neuroplasticity processes of PSD, comorbidities, swallowing screening and evaluation approaches, and swallowing rehabilitation modalities. We will examine the aforementioned hotspots and cutting-edge developments to give references and lessons for researchers in the field of PSD rehabilitation.

#### 4.2.1 Pathogenesis of PSD and mechanisms of neuroplasticity

According to data, the incidence of dysphagia in hemorrhagic stroke is between 58.6 and 67% (36, 37). The cortex is important in swallowing control, and subcortical lesion sites are usually associated with swallowing dysfunction. Even a small volume of hemorrhage can disrupt the neural network of swallowing, leading to swallowing difficulties, which is why hemorrhagic stroke patients have a high incidence of dysphagia (38). Dysphagia occurs in 32.3% of ischemic stroke patients, with subcortical infarction being more likely to develop moderate to severe dysphagia, and individuals with dysphagia generally have bilateral vertebral tract injury (39–41). According to research, bilateral subcortical lesions are major determinants impacting patient prognosis (42), and physicians must pay attention to this prognostic indicator to assess the patient’s prognosis. Furthermore, several studies have shown that patients with PSD have a poor neurophysiological response in the right hemisphere, dysphagia caused by infratentorial stroke is more severe than dysphagia caused by supratentorial stroke, and the right lenticular nucleus is associated with the development and severity of dysphagia (43–45).

According to multicenter retrospective cohort research, the majority of stroke patients have sarcopenia, and there is an independent negative link between sarcopenia and the recovery of swallowing function (46). Another research validated the link between temporal muscle thickness and dysphagia in stroke patients, demonstrating that temporal muscle thickness is an independent risk factor for dysphagia in patients with acute stroke (47). It is essential to note that significant muscular weakness and muscle volume loss can cause delayed dysphagia longer than 7 days after a stroke, therefore it is critical to recognize and diagnose early and execute early management (48). The foregoing findings open up new avenues for future study, and it is critical to assess if stroke patients have dysphagia caused by muscle loss to give early and personalized preventative and therapeutic approaches.

The brain is capable of neuroplasticity, and the cerebral cortex has an evident hemisphere response to swallowing. It has been discovered that individuals with recurrent cerebral infarction and central dysphagia exhibit compensatory remodeling of neurological function (49). Functional magnetic resonance imaging (fMRI) is a useful tool for studying the neurophysiology of swallowing *in vivo*, and it has been used to study the manifestations of the neural control of swallowing in normal subjects and patients with dysphagia, as well as the effects of swallowing therapy on neuroplasticity (50). Stroke is classified into four phases: hyperacute, acute, subacute, and chronic, with the window of greatest neural plasticity in the subacute phase also being a sensitive period for stroke recovery, and the potential for inducing recovery in the chronic phase becoming limited over time (51). Even though the chronic phase of stroke often lacks neuro independent recovery (52, 53), recovery is still achievable with intense neurorehabilitation, but at a significantly lower pace than in the subacute period (54). Functional connectivity between the cortex and the medulla was found to be enhanced in patients with episodic stroke, and functional connectivity between the cortex and the medulla could serve as a biomarker of swallowing-related changes in the neural network of the brain, implying that the brain’s precentral gyrus plays an important role in the regulation of neuroplasticity in pharyngeal swallowing (55). Increased excitability of the swallowing cortical bulb is primarily located in the undamaged cerebral hemisphere, and the sensory-driven human motor cortex is largely dependent on the frequency, intensity, and duration applied to promote neuroplasticity after brain injury (23), suggesting that there may be individual differences in the rehabilitation effects of PSD patients after using the same swallowing function rehabilitation intervention method. As a result, it is critical to analyze the patients’ degree of swallowing impairment and select the optimal intervention method to accomplish the therapeutic impact in the future rehabilitation process.

#### 4.2.2 Complications of PSD

Patients with PSD have a 4.35 times higher prevalence of pneumonia and are up to 4.07 times more likely to die than non-PSD patients, and the risk of PSD is strongly associated with hemorrhagic strokes, a history of previous strokes, severe strokes, gender (women are more likely to have a stroke than men), and diabetes mellitus (56). The severity of PSD modifies the patient’s food patterns, and alterations in eating pathways increase the likelihood of respiratory infections (57). According to studies, more than a quarter of patients with subarachnoid hemorrhage are not evaluated for dysphagia, and one-fifth of these patients acquire pneumonia (58) and delays in dysphagia screening and evaluation increase the risk of pneumonia infection. As a consequence, detecting and treating dysphagia in stroke patients at an early stage can help minimize the risk of pneumonia and death (2, 59). Training the respiratory muscles after a stroke can prevent infiltration or aspiration when swallowing fluid push, lowering the risk of respiratory problems and improving dysphagia (60). Malnutrition and insufficient fluid intake are common in PSD patients (61, 62), and malnutrition hurts stroke patients’ recovery of physical and swallowing function, prolonging hospitalization, increasing the burden of care, and increasing mortality. Nutritional screening and evaluation can help predict swallowing and physical function results in patients. As a result, there is a need for early nutritional evaluation and the adoption of early nutritional therapies that promote swallowing function recovery and enhance patient prognosis (63, 64). There is a risk of dehydration for patients after an acute stroke, particularly those with PSD, and optimal fluid replacement appears to be extremely important in reducing the risk of neurological deterioration and other complications; however, there is a lack of a clear method of hydration status assessment, a shortage of time for healthcare staff, unclear work patterns, and a low emphasis on hydration status management (65). Healthcare collaboration is critical in the treatment of hydration status after stroke, and there is a need to better define the stages and techniques of hydration status evaluation, as well as to raise healthcare personnel’s awareness of hydration status management to satisfy patients’ rehydration needs.

#### 4.2.3 Swallowing screening methods

Swallowing screening is one of the hottest subjects in the world of PSD rehabilitation research. Guidelines recommend that stroke patients be screened for swallowing before oral intake (such as medications, food, and liquids) and that abnormalities on initial screening be referred to a clinician trained in Speech-Language Pathology, Occupational Therapy, Dietitian, or Trained Dysphagia Clinician for a more detailed bedside para pharyngeal swallowing assessment and management of swallowing, feeding, nutrition, and hydration status (66). Among the swallowing screening evaluation procedures, the Water Swallowing Test (WST) and the Gugging Swallowing Screen (GUSS) are the most widely used and actionable. The Water Swallowing Test can determine whether a patient has significant malabsorption and whether instrumentation is required to further assess dysphagia to reduce unnecessary instrumentation; however, it is difficult to identify patients with silent malabsorption and carries the risk of false negatives (67). The GUSS is primarily indicated for acute stroke patients; the assessment method takes into account the case physiology of voluntary swallowing, and allows for the use of foods of varying consistencies in the swallow test to reduce the risk of aspiration in acute stroke patients due to difficulty swallowing liquids, has a high sensitivity (close to 100%), and is a reliable tool for the detection of dysphagia and the screening of patients at high risk of aspiration (68, 69). The Volume-viscosity swallow test (V-VST) is used to examine dysphagia using fluids and thickeners in conjunction with pulse oximetry. It may be used not only for screening but also to offer reliable indications of ideal fluid push volume and viscosity as well as therapy suggestions. V-VST, on the other hand, is reliant on the prevalence of the context or clinical situation in which it is used, with a positive predictive value of 68.3% (false positives as high as 31.7%) when the prevalence does not reach 30, and 95.2% in populations with a prevalence of 80% (70). Videofluoroscopy (VFS) and fiberoptic endoscopic evaluation (FFE) of swallowing are more accurate instrumental evaluations for assessing swallowing function and providing information on swallowing physiology, anatomy, and function. However, they have significant limitations in their use, as they are susceptible to patient posture, somatic status, cognition, and acceptance, secondly, the operation of VFS and FFE requires specialized equipment and training, and it is currently not possible to identify a swallowing screening tool that has high accuracy, sensitivity, specificity, and is perfect (8, 71), and a combination of approaches may be required.

#### 4.2.4 Rehabilitation for PSD

The emphasis of the keyword analysis study is neurorehabilitation. The most explored rehabilitative methods with the highest research enthusiasm are repeated transcranial magnetic stimulation (rTMS), transcranial direct current stimulation (tDCS), and acupuncture. rTMS is a non-invasive brain stimulation technique that reverses the pharyngeal motor cortex, modulates brain activity and swallowing motor behavior, and also modulates neurotransmitters, activates/polarizes immune cells (astrocytes and microglia), and inflammatory cytokines in the brain, thereby affecting brain function and improving post-stroke dysfunction (72, 73). A meta-analysis has shown that rTMS not only enhances swallowing function and daily life activities for PSD patients and reduces the incidence of aspiration but also proves to be a safe and feasible rehabilitation method (74), however, to accomplish excellent recovery, attention should be required to determine the proper stimulation intensity during the intervention. tDCS can improve dysphagia recovery after stroke by promoting reconfiguration of the swallowing neural network, so expediting dysphagia recovery after acute stroke, and the earlier therapy is started, the better the outcome of dysphagia recovery (75). A meta-analysis, however, found that tDCS was helpful for dysphagia after unilateral hemisphere stroke, medullary palsy, and brainstem stroke, but not for ataxia and dysphagia after basal ganglia stroke (76). Furthermore, because tDCS may be susceptible to the baseline level of the study population, such as age, affected cerebral hemispheres, comorbidities, and complications after acute stroke, attention should be paid to the program design of tDCS in rehabilitation. As a result, in the case of tDCS interventions, it is common to combine tDCS with motor-specific or peripheral sensory-motor stimulation and to combine cerebral stimulation with sensory feedback to entrainment (77). Furthermore, in terms of neurostimulation therapies such as neuromuscular electrical stimulation (NMES), pharyngeal electrical stimulation (PES), and transcranial direct current stimulation (tDCS), a combined approach would be more effective than single neurostimulation therapies or traditional dysphagia therapies in improving PSD (78). Acupuncture affects the bilateral cerebral hemispheres via feature brain areas of interest (ROIs), reduces aberrant functional connectivity, and enhances motor cortex recovery after stroke. By combining intervention with swallowing training, acupuncture can significantly boost the rehabilitative result (79, 80). Although evidence for the potential therapeutic alternatives and efficacy of acupuncture continues to emerge, international standards to assist clinical practice and treatment have yet to be produced (81).

In addition to these three hot rehabilitation modalities, intermittent theta burst stimulation (iTBS) is a non-invasive brain stimulation technique. By bidirectionally enhancing the excitability of the motor cortex associated with the genioglossus muscle and promoting neural remodeling, it effectively augments the swallowing function in patients with post-stroke dysphagia (82). It’s worth noting that, compared to rTMS, the combination of iTBS with swallowing exercises demonstrates a more pronounced improvement in the excitability of the swallowing motor cortex and swallowing function following a stroke. When combined with swallowing exercises that stimulate both cerebellar hemispheres, iTBS significantly reduces scores on the Fiberoptic Endoscopic Dysphagia Severity Scale (FEDSS), the Penetration-Aspiration Scale (PAS), and the Standard Swallowing Assessment (SSA). Concurrently, it notably elevates scores on the Functional Oral Intake Scale (FOIS), effectively improving swallowing function (83). Transcutaneous auricular electrical vagus nerve stimulation (ta-VNS) modulates swallowing function via bilateral extra-auricular stimulation of the vagus nerve, with few serious adverse events occurring during the intervention, which is safe and can be used as a novel non-invasive treatment strategy (84). Furthermore, the involvement of speech-language pathologists (SLPs) and nurses in the management of PSD rehabilitation is critical, and this co-management model can assist the rehabilitation team in consistently obtaining safety information about dysphagia and facilitating the implementation of the rehabilitation program; however, implementable options are needed to be explored further (85).

## 5 Strengths and limitations

The bibliometric analysis demonstrates significant differences and advantages over other online tools in the realm of academic research. Firstly, bibliometric analysis is specifically tailored to peer-reviewed scholarly articles, ensuring depth, breadth, and high quality of data. This level of specificity allows researchers to gain a comprehensive understanding of a research field, a depth that many online tools often fall short of. Additionally, it offers analyses spanning extended timeframes, making it possible to reveal evolving research trends, a feature not easily found in other tools. Using tools such as CiteSpace and VOSviewer for bibliometric studies enables an in-depth exploration of relationships among research topics and provides a structured overview. Concurrently, it relies on standardized academic databases, ensuring the data’s scientific integrity, accuracy, and consistency. This mode of analysis also offers a quantitative approach for researchers to evaluate the impact and significance of their work and aids in unveiling patterns and trends in research collaborations. While some online tools may boast advantages in real-time updates, visualization, or user interface, bibliometric analysis indisputably excels in depth, scope, and scientific rigor. Therefore, to provide a more comprehensive and systematic insight, we employed the bibliometric analysis approach to study literature related to post-stroke dysphagia rehabilitation, culminating in a dedicated research article.

Using the CiteSpace, VOSviewer, and Bibliometrix R software packages for bibliometric analysis, this study synthesizes the strengths of the three software packages to provide an overview of the research progress and cutting-edge trends in the field of PSD rehabilitation around the world, allowing researchers to quickly understand the current state of research and hotspots in the field. However, because this study is not a substitute for a systematic review, there are certain limitations to this review. First, due to the limitations of the software and research methodology, we only selected literature from the WoSCC database, whereas most other databases, such as PubMed, Embase, and Scopus, do not have comprehensive information about their full text and citation analysis, which is why we chose the WoSCC database. As a result, this may neglect the contribution of literature from other databases in this study field. Second, we merely screened the retrieved literature based on the inclusion and exclusion criteria and did not analyze the quality of the literature, which might be biased. Third, certain high-quality publications may be ignored in the literature analysis process due to their recent publication and low citation counts, which may contribute to bias. However, the WoSCC database has a huge amount of high-quality core literature from all around the world, and we continue to feel that our study covers the research hotspots and frontiers of PSD rehabilitation and can give certain information for future research.

## 6 Conclusion

In this study, the WoSCC database was searched for the literature on PSD rehabilitation from the previous 25 years, and the titles and abstracts of the literature were read and screened according to the inclusion and exclusion criteria, and those that met the criteria requirements were included as required. The study of the number of publications, nations, institutions, authors, co-cited references, and keywords using the CiteSpace, VOSviewer, and Bibliometrix R software packages explains the general picture of PSD rehabilitation research and highlights research hotspots and cutting-edge trends. The visualization analysis findings reveal that research on this topic is gaining more and more attention from researchers, the number of publications is increasing, and the research field is in the process of continuous exploration. According to the study, future research hotspots and frontiers will mostly cover the pathophysiology and neuroplasticity processes of PSD, comorbidities, swallowing screening and evaluation methodologies, and swallowing rehabilitation modalities. These research goals and frontiers underscore the significance of PSD management in the swallowing rehabilitation process. Overall, this work may successfully give a research trajectory of PSD rehabilitation as well as significant research information and research suggestions to researchers interested in this subject.

## Author contributions

YH: Data curation, Formal analysis, Software, Visualization, Writing – original draft. XT: Data curation, Formal analysis, Software, Visualization, Writing – original draft. HK: Writing – review & editing. HW: Data curation, Software, Writing – review & editing. YX: Writing – review & editing. DZ: Supervision, Writing – review & editing. CL: Supervision, Writing – review & editing.
